# Aptamer-Based Diagnostic Systems for the Rapid Screening of TB at the Point-of-Care

**DOI:** 10.3390/diagnostics11081352

**Published:** 2021-07-28

**Authors:** Darius Riziki Martin, Nicole Remaliah Sibuyi, Phumuzile Dube, Adewale Oluwaseun Fadaka, Ruben Cloete, Martin Onani, Abram Madimabe Madiehe, Mervin Meyer

**Affiliations:** 1 DSI/Mintek Nanotechnology Innovation Centre-Biolabels Node, Department of Biotechnology, University of the Western Cape, Private Bag X17, Bellville 7535, South Africa; 3517594@myuwc.ac.za (D.R.M.); nsibuyi@uwc.ac.za (N.R.S.); phumuedube@gmail.com (P.D.); afadaka@uwc.ac.za (A.O.F.); amadiehe@uwc.ac.za (A.M.M.); 2 South African Medical Research Council Bioinformatics Unit, South African National Bioinformatics Institute, University of the Western Cape, Private Bag X17, Bellville 7535, South Africa; ruben@sanbi.ac.za; 3 Department of Chemistry, University of the Western Cape, Private Bag X17, Bellville 7535, South Africa; monani@uwc.ac.za

**Keywords:** tuberculosis, aptamers, diagnostics, lateral flow assays, point-of-care, biomarkers

## Abstract

The transmission of Tuberculosis (TB) is very rapid and the burden it places on health care systems is felt globally. The effective management and prevention of this disease requires that it is detected early. Current TB diagnostic approaches, such as the culture, sputum smear, skin tuberculin, and molecular tests are time-consuming, and some are unaffordable for low-income countries. Rapid tests for disease biomarker detection are mostly based on immunological assays that use antibodies which are costly to produce, have low sensitivity and stability. Aptamers can replace antibodies in these diagnostic tests for the development of new rapid tests that are more cost effective; more stable at high temperatures and therefore have a better shelf life; do not have batch-to-batch variations, and thus more consistently bind to a specific target with similar or higher specificity and selectivity and are therefore more reliable. Advancements in TB research, in particular the application of proteomics to identify TB specific biomarkers, led to the identification of a number of biomarker proteins, that can be used to develop aptamer-based diagnostic assays able to screen individuals at the point-of-care (POC) more efficiently in resource-limited settings.

## 1. Introduction

Strategies and policies to eradicate TB have been set by the World Health Organization (WHO) [1] and the key factors to achieving these goals are through early diagnosis and treatment of the disease. Diagnosis of active TB can only be confirmed when there is a definite presence of the causative agent, *Mycobacterium tuberculosis* (*M. tb*), in the patient’s body. However, the prompt and accurate diagnosis of TB still remains a significant medical challenge, particularly in poor resource settings [2]. Commonly used TB diagnostic techniques include sputum culture, microscopy, and molecular tests. The sputum culture test has been in practice for over a century, and it is still regarded as the standard test for TB diagnosis [3,4].

The test has a number of limitations; it detects all acid-fast bacilli in sputum samples and is not specific for *M. tb* [3], has a long turnaround time of 4–8 weeks and is not suitable to diagnose TB in children under the age of 5 years [5,6]. More specific molecular tests such as the GeneXpert and line probe assays are available but are quite expensive for low resource settings as they require specialised equipment and highly trained personnel to perform the tests [7]. More cost-effective rapid lateral flow immunoassays for the diagnosis of TB are available, but these tests often have poor sensitivity and specificity that stems from the low stability of antibodies in harsh environmental conditions [8]. To combat TB, more effective, rapid, sensitive, and cost-effective TB diagnostic strategies are urgently required. Replacing antibody-based diagnostic systems with aptamer-based systems has the potential to overcome the challenges associated with existing immunological assays [9]. Aptamers are short single-stranded nucleic acids that bind to specific target molecules by folding into specific structures [10]. Aptamers fold into specific 3D conformations that provide the structural specificity required for binding to target molecules through shape complementarity, electrostatic interactions, π–π stacking interactions, and hydrogen bonding [10]. Highly specific aptamers have been developed against a diverse range of targets, which include cells, viruses, proteins, drugs, and metal ions [11,12]. They can be produced synthetically at a large scale, and therefore have significantly lower production costs when compared to antibodies [13]. The use of aptamers in development of TB diagnostic devices that are based on the lateral flow assay may result in the production of highly specific and sensitive TB diagnostic kits [14].

### 1.1. Epidemiology of TB

TB is a major threat to human health due to rapid and ease of transmission. Despite the measures that are in place to eliminate TB, TB remains problematic due to several factors such as inadequate therapy, as well as late and missed diagnoses. Although *M. tb* is the main causative agent of TB, infections can also be caused by *Mycobacterium Bovis* (*M. bovis*), which is present in domesticated animals such as dogs and cattle [15,16]. In fact, up to 2000 of the TB cases that were reported in the U.S./Mexico border between 2006 and 2013 resulted from animal to human transmission [17].

The rate of infections is not slowing down, 10 million cases and about 1.4 million TB fatalities were reported worldwide in 2019 [1,18]. Of the reported deaths, about 95% occurred in low to middle-income countries [1,19]. Men are more susceptible to TB infections than women, and can significantly affect fertility in women [19,20]. The burden of TB varies among countries, least affected countries report as low as 5 new TB cases per 100,000 population, while highly affected countries report more than 500 new TB cases for the same population size [1].

TB is the predominant comorbidity of HIV/AIDS, due to the fact that HIV-positive (HIV^+^) patients are immunocompromised and thus more vulnerable to infections such as TB [20]. In 2019, about 209,000 TB patients were also co-infected with HIV (TB-HIV^+^) globally of which 14,000 were diagnosed with Multidrug-Resistant TB (MDR-TB) [1]. The mortality rate for TB-HIV^+^ and TB-HIV^−^ patients in 2019 were estimated at 62 and 38 cases per 100,000 population, respectively [1]. There is compelling evidence that shows that TB preventive therapy can help curb the disease; however, such therapeutic approaches are not commonly offered in resource-limited countries. About 35% of *M. tb* infected people are not accurately diagnosed and do not receive the appropriate treatment due to the unavailability of healthcare facilities. It is estimated that in SA this contributed to the death of approximately 36,000 HIV^+^ and 22,000 HIV^−^ patients in 2019 in SA [1]. In addition, there was an estimated 182,000 deaths due to MDR-TB, which is thought to result from lengthy anti-TB treatment and sporadic drug shortages are also a major concern [21]. When left untreated, 10% of MDR-TB patients can develop extensively drug-resistant TB (XDR-TB) within the same year of diagnosis [7]. Hence, preventive healthcare measures are recommended by the WHO to reduce the risk of TB infections, especially among HIV^+^ individuals [1]. Sustainable Development Goals (SDG) and End TB Strategy are two of the strategies supported by the WHO aimed at reducing and ultimately eliminate TB. Based on the current TB infection rates it might not be possible to attain the goals set by the SDG, i.e., to reduce global TB incidence rates by 80% (including new and relapse cases) by 2030 [1,22]. Similarly, the goals set by the End TB Strategy, which aims to decrease the incidence rates to 83 cases per 100,000 population size by 2035, may also not be achieved [1,23,24]. Although the number of TB cases has gradually decreased over the years, its global burden is still hefty [1,25] and urgent steps need to be taken towards the development of new diagnostic methods, vaccines, and effective treatments for TB [26]. Effective TB diagnosis and treatment haves saved millions of lives of both HIV^−^ and HIV^+^ TB patients between 2000 and 2019 [1,22] and is possibly the only hope towards eradication of TB.

### 1.2. Stages of TB Infection and Consequences

**Figure 1:**
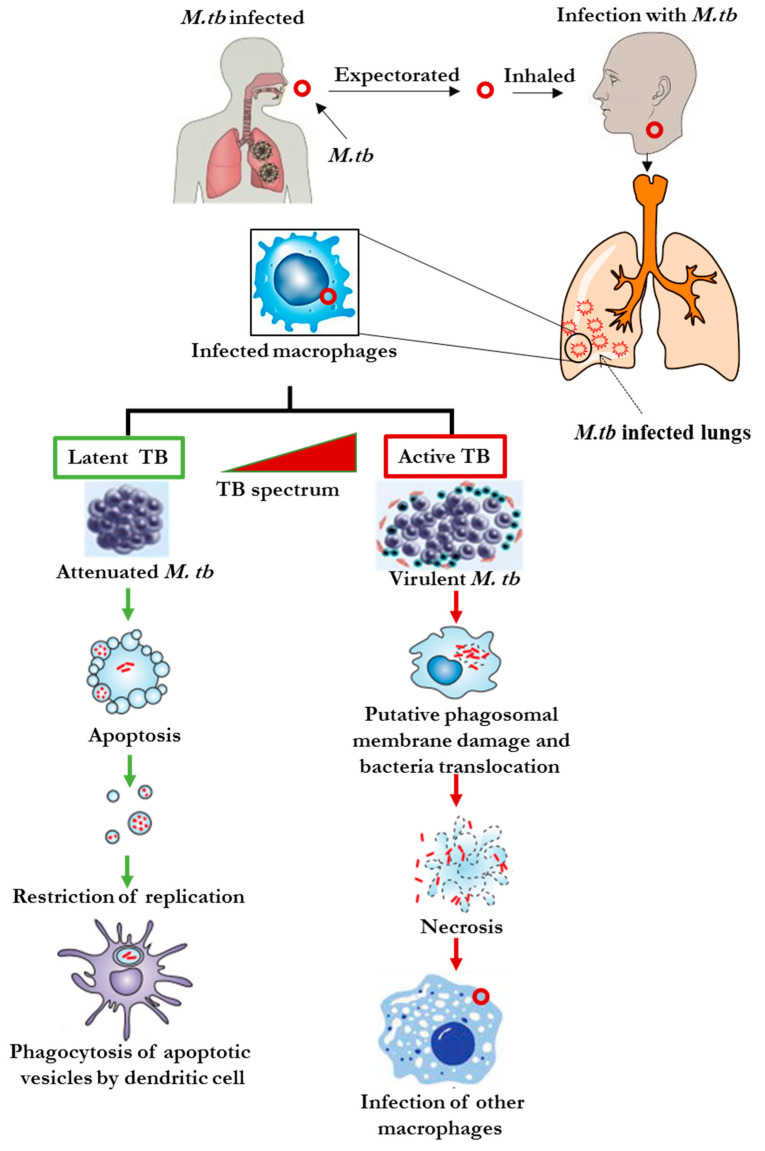
Stages of the TB infections, depicting the latent and active stages.

*M. tb* enters the body mainly via inhalation of aerosols containing bacterial particles, which are then transported into tissues, mainly the lungs. After the *M. tb* reach the alveoli in the lungs, the bacilli are ingested by the alveolar macrophages that prime them for destruction or suppress their growth [27]. In individuals with a competent immune system, the bacteria will be engulfed by the macrophages. At this stage, the bacterial replication is stopped, and the invading microorganisms are destroyed. If the *M. tb* is not destroyed, the infection can take one of two possible paths, either latent or active TB infection [28] as shown in Figure 1.

In a latent TB infection, *M. tb* remains in an inactive state in the immune cells for prolonged periods of time. Latent infected individuals do not develop TB disease due to robust T cells that trigger apoptosis or programmed cell death of *M. tb* infected host cells. While they do not develop the disease, latent infected individuals will have a positive reaction to the tuberculin skin test or TB blood test [29]. When the infected individual’s immune system becomes weakened and can no longer contain the latent bacteria, reactivation of latent TB may occur, the bacteria becomes active, and the infected person will develop symptoms of TB. During active TB, *M. tb* is able to replicate rapidly, eventually spreading to other parts of the body [30]. Infection with active TB promotes accumulation of inflammatory cytokines, including interleukin-17 (IL-17) and interferon gamma (IFNγ), which trigger necrosis, resulting in the cavitation of the infected area, in the lungs [31].

The infection is spread through the lymphatic system or to distant tissues and organs such as regional lymph nodes, the apex of the lungs, kidneys, brain, larynx, pleura, joints, and bones. At this stage, the latent *M. tb* becomes active and the person develops active or virulent TB [32], as depicted in Figure 1. Individuals who are immunocompromised, as in the case of HIV^+^ persons, have been reported to have an increased risk of latent TB reactivation [33]. The risk of reactivation is estimated to be 5–10% for a latent infected individual, with the majority of latent infected individuals developing active TB within the first five years after initial infection [34]. Active TB infections occur when the immune system is not competent to defend against the invading *M. tb* [35]. Some people may develop active TB within a few weeks of infection, while for others it may take years [36]. About 90% of the active TB cases are due to the reactivation of latent TB infections [35]. However, some people with latent TB infections may never develop active TB in the course of their lifetime [36]. Infection with *M. tb* either causes Pulmonary TB (PTB), Extra Pulmonary TB (EPTB), or TB lymphadenitis [37]. In PTB, the infection occurs at the upper area of the pulmonary lobe of the lungs and is considered highly contagious. PTB patients usually have abnormal chest radiography as well as a persistent cough. While in the majority of TB cases the lungs are the affected organ, some TB infections can evade the immune and spread to other anatomical sites [37,38] giving rise to EPTB [38]. EPTB affect different organs outside the pulmonary system [39]. The infection might also spread to the central nervous system, resulting in TB meningitis, which could be fatal if left untreated [39,40]. In cases where a patient is co-infected with HIV, the patient could develop either PTB or EPTB [41]. EPTB can be classified as Miliary TB, TB Meningitis or TB lymphadenitis. Miliary TB is a serious but rare disease where the bacterial particles enter the bloodstream, replicating and manifesting in multiple organs of the body. This type of TB is common in children up to the age of 14 years and adults that are severely immunocompromised [37,39]. TB meningitis is normally diagnosed by imaging the base of the brain. The symptoms include stiffness in the neck, headaches, and loss of consciousness [37,39]. These symptoms, however, can be mistaken for Miliary TB which has similar symptoms and produce similar chest radiographs to that of TB meningitis [39]. TB lymphadenitis (Lymph node infections) usually occurs in children below the age of 14 years and cause enlargement of one or more lymph nodes [39,42]. In adults, *M. tb* thrives mainly in the lungs rather than the lymph nodes. In fact, PTB occurs in ±80% of TB patients while lymph node infections caused by *M. tb* arises in only ±20% of active TB patients. The risk of lymph node infections is high in immunocompromised individuals such as those co-infected with HIV [22].

### 1.3. The Mycobacterium Tuberculosis Complex (MTBC)

While *M. tb* causes TB in humans, other *Mycobacterium* species such as *M. africanum, M. caprae, M. bovis, M. microti, M. mungi, M. pinnipedi* and *M. canetti* are known to cause TB in animals. The *Mycobacterium* genus consists of over 190 species that share similar characteristics, which include immobility, mycolic acids-rich thick cell wall, aerobic and acid-fast nature [27], and can be distinguished based on species-specific markers. These arise either from single base mutations or combinations of polymorphisms [43].

#### *M. tb* Strains

The persistence of TB infections is fuelled by the emergence of new strains of *M*. *tb*. The emergence of MDR and XDR *M. tb* strains has impeded the eradication of TB [1,34,39]. To alleviate the increasing prevalence of TB, the Bacillus Calmette–Guérin (BCG) vaccination was introduced [1,44]. The *M. tb* strains are believed to have evolved alongside the ancient hominids, and their evolution continues to date. Phylogenetic analysis of the *Mycobacterium* genome identified seven different lineages of *M. tb* strains linked to a particular geographic location [45,46].

Lineage 1 is most prevalent in some parts of East Africa, the Indian Ocean rim, and the Philippines. Lineage 2, which includes the Beijing strain, is prevalent in East Asia while lineage 3 is endemic in some parts of East Africa as well as the Indian subcontinent. Lineage 4 includes the Latin American-Mediterranean (LAM), X type, Harlem and T families and is widespread in America, the Middle East, Europe, and some parts of Africa. Lineages 5 and 6 have been identified in West Africa, while Ethiopia and part of East Africa, have reported more cases of lineage 7 among the Djibouti immigrants [45,46]. The Beijing strain is more likely to acquire drug resistance when compared to other strains [47]. Additionally, both the Beijing and Indo-Oceanic strains (lineage 1) were associated with TB Meningitis [48]. Strains in Lineage 3 have the lowest transmission rate when compared to other lineages [49]. In Ghana, *M*. *africanum* (lineage 5) acquires drug resistance at a slower rate than the Euro-American strains (lineage 4) [50]. The Beijing strain has been identified as a major cause of drug resistant TB infections, while the LAM4/KwaZulu-Natal (KZN) strains caused MDR and XDR-TB in SA [51,52,53]. The KZN province in SA has reported the highest incidence and mortality rate of MDR and XDR-TB mainly caused by the LAM4/KZN strain [54]. LAM4/KZN strain is part of the Euro-American strains first identified in KZN in 1994, the reasons for the high prevalence in KZN remains unclear [54]. In other areas of SA such as the Eastern and Western Cape provinces, the Beijing strain still remains the most prevalent TB causing strain [55,56]. The hyper-virulent nature of the Beijing strain could elucidate the prevalence of the strain to evade host defences and invade human alveolar [47,57].

### 1.4. Clinical TB Diagnostic Techniques and Their Limitations

**Figure 2:**
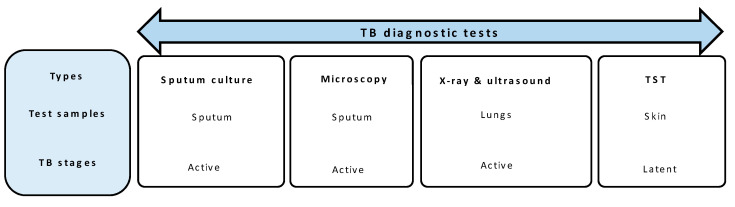
Widely used clinical TB diagnostic tests.

*M. tb* is a group 3 risk agent that falls under Biosafety Level 3 (BSL3) for laboratory testing [58]. A number of clinical TB diagnostic tests are available to detect the *M. tb* infections as discussed below. Details of widely used TB diagnostic tests are shown in Figure 2. The standard tests in low resource settings are the sputum smear microscopy and sputum culture tests. The sputum smear microscopy test is often used in countries with a high rate of TB infections to detect the presence of *M. tb* either by light (Ziehl–Neelsen stain) or fluorescence (acridine–orange stain) microscopy [59]. The sputum culture test is also used as a confirmatory test for samples that test negative in rapid biomarker-based screening tests [60]. The sputum smear test is easy to perform, provides results within a few hours, and is generally more cost effective when compared to the sputum culture test [60]. However, the sensitivity of the sputum smear test is about 50–60% [59,61], especially in countries with high TB and HIV co-infections [62]. The sputum culture test is time consuming (~4 weeks turn-around time) and unable to detect *M. tb* in the early stages of infection [63,64]. Specialised equipment and level 3 biosafety laboratories are also required to safely perform sputum culture tests.

*M. tb* primarily infects the lungs and then spreads to other parts of the body. When the lungs are infected, the immune cells trigger an inflammatory response that can cause damage to the lungs and other organs. The lungs gradually develop scar tissue which can be visualised using a chest X-ray or a lung ultra-sound [65,66]. However, these techniques are unable to distinguish between lesions caused by other lung diseases, such as lung cancer and pneumonia, that are unrelated to TB [67]. Thus, a negative chest X-ray result does not confirm that the patient is EPTB negative. Hence, chest X-ray results are always be confirmed using other TB diagnostic tests. Although the turn-around time for chest X-ray test results is relatively short in comparison to the other TB tests, the test requires specialised equipment and trained personnel [68,69].

The Tuberculin Skin Test (TST) is used to assess whether a person has been exposed to *M. tb*, either through a previous TB vaccination or environmental exposure. After an immune competent individual is exposed to *M. tb,* an active immune response is triggered resulting in the production of antibodies as well as memory B lymphocytes that will recognize *M. tb* antigens upon re-infection and produce *M. tb* antibodies to fight off future TB infections. TST involves the injection of a small amount of tuberculin fluid containing *M. tb* protein antigen, Purified Protein Derivative, PPD, into a patient’s forearm. After injection, the PPD reacts with the pre-existing antibodies and inflammation causes visible swelling of tissue at the site of injection within 48–72 hrs [70]. The larger the swelling on the skin, the higher the likelihood that the person has been previously exposed to *M. tb*. However, TST is unable to distinguish between the latent TB and active TB. The TST will also produce a positive result for individuals who have been vaccinated against TB with the BCG vaccine [70].

#### 1.4.1. TB-Specific Diagnostic Tests

The principle of molecular diagnostic tests involves the detection of genomic, proteomic or metabolomic markers that can be associated with the disease. These diagnostic tests are often used as conclusive TB diagnostic tools but can also be employed as prognostic tools to predict the outcome of treatments [71].

##### Interferon Gamma Release Assays (IGRAs)

Interferon gamma (IFN-ƴ) release assays (IGRAs) are considered more accurate than the TST as they measure a patient’s immune response to *M. tb* [72]. Blood samples taken from individuals suspected of being infected with *M. tb* is exposed to *M. tb* antigens [73] and if the individual is TB-positive, their peripheral blood lymphocytes respond by producing IFN-ƴ. These assays are considered to be highly sensitive, and a single test can give a conclusive TB diagnosis. The US Food and Drug Administration (FDA) approved two more IGRAs: the T-SPOT^®^ TB test (T-SPOT) and the QuantiFERON^®^ TB Gold In-Tube test (QFT-GIT) [73]. The QFT-GIT test measures the IFN-ƴ concentration, whilst the T-SPOT determines the number of cells actively producing IFN-ƴ. A summary detailing comparison of the two tests is given in Table 1.

Despite their increase in sensitivity, these tests are susceptible to errors which can be introduced during sample collection, transportation, processing, and data analysis. The tests must be done within 8–30 h of sample collection, whilst the lymphocytes are still viable. The high cost of these tests makes them unaffordable in low-resource settings. Although considered accurate, these tests have reduced accuracy in individuals co-infected with HIV, children younger than 5 years, and individuals previously diagnosed with TB. Furthermore, these tests are unable to fully differentiate between latent and active TB infections [7]. From 2011 to 2014, the average cost for a single T-SPOT and QFT-GIT test was USD 46.61 and 55.08, respectively [74].

##### GeneXpert Test

GeneXpert is a cartridge-based, automated and rapid molecular diagnostic technique that performs sample processing and hemi-nested real-time PCR analysis in a single, hands-free step. It is also used to identify genetic mutations in *M. tb* responsible for resistance towards antibiotics such as rifampicin [75]. Due to its high sensitivity, specificity, and ability to detect DNA mutations responsible for antibiotic resistance, the test is ideal for the diagnosis of MDR-TB. The GeneXpert test has an overall sensitivity of 98.6%, specificity of 100%, positive prediction value of 100% and negative prediction value of 93.8% [76]. GeneXpert test is able to detect *M. tb* DNA in a sputum sample within 2 h [77].

This test was first launched in 2008; SA and India were among the countries chosen to perform the clinical validation of this test [5]. Despite the necessity of diagnostic approaches such as GeneXpert in countries with high TB incidences, the cost is considered exorbitant. The unit cost of a single GeneXpert test in the USA was about USD 12.9 in 2018 [78]. However, based on 2018 estimates, this technology is too costly for developing countries as the average testing and cartridge costs were reported to be USD 113 and 10.7, respectively [78]. Nevertheless, over 150 civil society organisations across the world have joined ‘The Time for 5 US$ Coalition’, which aims to reduce the cartridge costs to USD 5 [79].

Although considered highly reliable, the accuracy of the GeneXpert is also affected by co-infection with HIV and the test cannot differentiate between latent and active TB infections [80]. Furthermore, the test requires highly trained personnel, as well as a level 3 biosafety laboratory facility [76]. Regardless of all these limitations, the GeneXpert test is currently the best performing TB diagnostic test and is therefore used as the gold standard for the detection of antibiotic resistant TB. When assessing possible treatment outcomes, the GeneXpert test is more accurate and has a rapid turn-around time when compared to the conventional drug susceptibility tests [76].

Little et al. compared the cost and efficiency of IGRA tests against GeneXpert and sputum smear tests in India and concluded that the use of IGRA tests to diagnose TB in a developing country such as India should be discouraged since the higher costs associated with this test does not necessarily benefit the health care systems of such countries [81]. While the use of the IGRA test identified 23,700 and 400 additional TB positive tests in comparison to sputum smear and GeneXpert tests, respectively, it also identified 315,700 and 70,400 false positive tests. The consequence of this is overtreatment and wasteful expenditure of limited resources.

##### Line Probe Assays

Line probe assays incorporate both PCR and reverse hybridisation techniques to rapidly detect *M. tb*, as well as to identify possible genetic mutations associated with *M. tb* resistance to rifampicin and isoniazid. Similar to the GeneXpert test, line probe assays provide rapid results and require both biosafety level 3 laboratory and highly trained personnel. Since line probe assays employ open-tube formats, the test samples are exposed to external environments, which means that the probability of sample contamination is much higher with this test [82]. Line probe assays also have high operational and maintenance costs. A single test is valued at USD 23.46 [83].

#### 1.4.2. Serological Tests for TB

Serodiagnostic tests are based on Lateral Flow Immuno (chromatographic) Assays (LFIAs) used to detect host anti-*M. tb* antibodies present in blood samples [84]. However, these antibody-based diagnostic tests have low sensitivity and specificity which results in false negative and positive results. Nevertheless, these tests are more cost effective compared to most other molecular assays.

WHO reported that more than a million serological tests that cost between USD 10 and 30 per test were carried out annually in India [85]. About 60,000 of these serological TB tests were conducted per month in India alone, and their summary cost was estimated to be USD 1.5 million per year [86]. This cost was estimated to be USD 15 million per year [86]. Despite the overall affordability of serological tests, WHO cautioned against their use in the diagnosis of active TB due to inconsistencies in the results [84,85].

Point of care (POC) rapid diagnostic tests have been produced to improve the control of TB infections. These tests can be carried out at primary healthcare facilities at the time of or near places of patient care, and results are generated immediately. A patient visiting a primary healthcare facility can be tested for TB immediately, and if diagnosed either receive the required treatment immediately or be referred for additional testing, if necessary [87,88,89]. POC diagnostics are defined as medical diagnostic tests and are mainly used for screening purposes rather than diagnostics [87,88,89,90]. An example of such a test is the commercially available lateral flow urine lipoarabinomannan assay (LF-LAM), which is used to screen for the presence of LAM protein in patients with active TB patients who are also co-infected with HIV [91]. These tests are cost effective and do not require expensive laboratory equipment or highly trained personnel. The generated test is usually easy to interpret and this enables its use as self-screening tests. With this in mind, POC diagnostic tests such as lateral flow assays (LFAs) are ideal for low resource settings [88,89], which bodes well for WHO’s ASSURED requirements (Affordable, Sensitive, Specific, User-friendly, Robust and rapid, Equipment-free, and Deliverable to those who need them).

**Figure 3:**
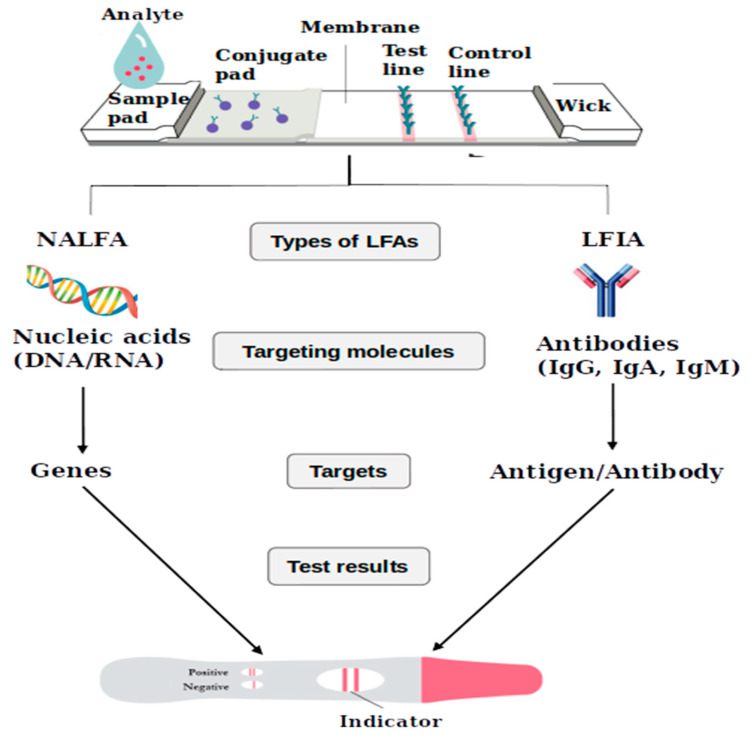
Types of LFAs employed in the detection of molecular targets associated with diseases in biological samples. NALFA detects the presence of nucleic acids while LFIA detects antibodies.

LFAs can be categorised as either LFIAs or nucleic acid lateral flow assays (NALFAs) depending on the recognition element used in the assay, as illustrated in Figure 3. The LFIAs use antibodies as recognition elements while the NALFAs use nucleic acid probes for the detection of amplicons obtained from PCR [92]. These tests are gaining popularity as more companies are developing lateral flow devices for the rapid diagnosis of infectious diseases, including TB [93].

Between 1990 and 2020, more than 100 companies worldwide have developed LFA devices for the rapid diagnosis of diseases with improved performance. The annual increase in production of lateral flow devices was compounded at 7%, with the global market for this technology estimated to reach USD 4.68 trillion by 2030 [93]. With respect to TB diagnostics, LFAs that are used for the diagnosis of TB have limited sensitivity (43–71%) and show significant inconsistency (84–100%) [94,95]. While several TB diagnostic tests have been developed and are currently used in clinical settings, these tests differ significantly in terms of their sensitivity, specificity, cost, and turn-around time as summarised in Table 2. Although GeneXpert and Line Probe assays have higher sensitivities and specificities, they are more costly compared to serological LFA tests, sputum tests, and IGRAs. The antibodies used in LFIAs are the limiting factor. This is due to the low stability of antibodies, posing a major challenge in the development of LFIAs [96]. Considering these limitations, the development of rapid, cost-effective, and sensitive POC diagnostic devices capable of diagnosing TB at an early stage of infection are urgently needed. In addition, such POC diagnostic devices should ideally also be able to differentiate between latent and active TB, as well as between TB infections and pulmonary conditions caused by factors other than TB. These enhancements will consequently improve the treatment outcomes and prognosis of TB patients. The application of aptamers as recognition elements in the LFAs can potentially lead to the development of such POC diagnostic devices.

### 1.5. Aptamer-Based Diagnostic Systems for Rapid Detection of TB

The term aptamer comes from two words, “*aptus*” which is Latin for “to fit”, and “*meros*” which is Greek for “part” or “region” [101,102,103]. Aptamers are synthetic single-stranded oligonucleotides (DNA or RNA) or peptide molecules that bind to a specific target [104]. They are shorter than 100 nucleotides (nts) in length and have an increased binding affinity and selectivity for their specific targets, ranging from small molecules such as metabolites and proteins to whole cells [105,106]. Their high binding affinity is attributed to the fact that they can fold into three-dimensional structures such as stems, internal loops, purine-rich bulges, hairpin structures, pseudo-knots, kissing complexes, and G-quadruplex structures [96]. Aptamers are able to discriminate between targets that only have subtle structural differences [107].

Antibodies have been used extensively in the development of rapid immunodiagnostic systems, but aptamers are emerging as alternative molecules with superior properties to antibodies, as summarised in Table 3 [96]. Aptamers are chemically synthesized in the laboratory, and they can be enriched to increase their target specificity and binding efficiency. Several SELEX methods used to date include Cell SELEX, Nitrocellulose membrane filtration-based SELEX, Affinity-based chromatography, Capillary electrophoresis-based SELEX, Microfluidic-based SELEX, Magnetic bead-based SELEX, and tailored-SELEX [108,109]. Most recently through the development of in silico approaches, a full set of in silico methods have been applied to select for best binding aptamers using docking tools [110]. The aptamers are then subjected to molecular dynamics (MD) to evaluate stability of aptamer/ligand complexes, followed by statistical analysis for binding evaluations and selection of the best binders for downstream applications [110].

While the identification of antibodies can a time-consuming process, the identification of highly specific aptamers can be relatively quick through in vitro or in vivo selection methods at a comparatively low cost. Most importantly, aptamers can be produced at large-scale using synthetic processes and it is possible to easily modify aptamers for biomedical applications. Their ability to tolerate high temperatures makes them appealing for use in development of diagnostic devices that are more effective and will have a longer shelf life [96,107]. 

### 1.6. Applications of Aptamers in the Diagnosis of Infectious Organisms

Over the past years, aptamers have proven their worth in various biomedical applications as targeting moieties. The afore-mentioned properties (Table 3) make them well-suited for use in the development of rapid and cost-effective POC devices such as lateral flow and colorimetric assays for the detection of disease biomarkers. The aptamer-based systems proved to be effective, sensitive, and accurate for detection of various pathogenic microbial agents such as bacteria, viruses, and protozoan parasites [112]. These included bio-related substances that differ slightly [113,114]. Their efficiency can be increased by developing a multiplex aptasensors which are able to detect multiple targets or biomarkers simultaneously [112,114]. The efficiency of the aptamer-based systems was comparable and sometimes superior to the standard tests such as PCR and ELISA. These systems were compatible with all types of samples including live samples [115,116].

#### Aptamers for Detection of *M. tb* Biomarkers

Biomarkers are signature molecules within the body fluids (urine, blood, saliva, and tears) and tissues whose presence can serve as an indicator of a particular biological condition or disease [117]. Biomarker levels can be used to classify patients according to the extent of the disease [118] and is the first step towards the prescription of relevant treatments [119]. Biomarkers are therefore key to understanding the disease state, the effect a particular disease has on the patient, determine the success of a specific treatment and are useful for the development of disease-specific theranostics [119,120].

Recent efforts in the field of TB diagnostics have revealed some of the TB biomarkers that have the potential for development of more TB-specific, rapid, efficient, and affordable diagnostic tools. TB infections are associated with multiple bacillary subpopulations that have distinct biosynthetic and metabolic profiles and anatomic localization [121] that can distinguish *M. tb* from *M. tb*-related species.

Molecules which include metabolites, DNA and protein, that originate from the intracellular, extracellular or cell surface of *M. tb* can be used as potential targets for TB diagnosis in various assays. Promising TB targets include the *M. tb* virulence factors (FbpA, FbpB, and Fpb) [122], *M. tb* specific proteins (phosphate-binding transporter lipoproteins PstS1) [123], extracellular *M. tb* antigens (MPT64 and MPT51) [124,125], inner membrane *M. tb*-specific protein (α-Crystalline; Acr and HspX) [126], and soluble *M. tb* proteins (CFP-2, -10, -30 and ESAT-6) [127,128]. However, some of these proteins are not specific to *M. tb* as they have also been identified in other *Mycobacterium* species [124,125]. Mycolic acids (Mas) were identified as ideal TB diagnostic markers [129] as they are involved in the first-line recognition of *M. tb* by the host immune cells and also protect the *M. tb* against host immune cells, such as the macrophages [129]. Some limitations associated with the use of Mas in TB diagnosis include the poorly understood mechanisms by which the Mas are transported within the body and cleared by the lungs. As biomarkers Mas have low sensitivity, are non-specific and are also present in non-tuberculous mycobacterial isolates (MAC Q14, MAC M151, *M. gordonae*, *M. fortuitum*, *M. simiae*, *M. kansasii*, *M. abscessus*, *M. chelonae*, and *M. xenopi*).

Aptamers against immuno-dominant antigens of the *M. tb*, such as CFP-10, ESAT-6, and CFP-10-ESAT-6 heterodimer have been identified and investigated for their detection of active TB in clinical sputum samples [127,128]. However, these aptamers have not been applied in any practical diagnostic tool due to their lack of specificity and sensitivity [130]. Mozioglu and colleagues selected CFP-10, ESAT-6, and the H37Rv aptamers using two different SELEX selection protocols and showed higher binding affinity of the aptamers to *M. tb* H37Ra than *M*. *bovis* and *E*. *coli* [131]. The H37Rv aptamers selected by Chen et al. had higher dissociation constants (K_D_ = 5.09 ± 1.43 nM) than those described by Mozioglu et al. [131,132]. The H37Rv aptamers inhibited the bacterial invasion of macrophages while decreasing bacterial growth in the lungs, thus demonstrating the dual function of this aptamers as both a TB diagnostic and an anti-TB agent [132].

DNA aptamers have also been employed in the development of *M. tb* diagnostic tests that targets the HspX TB biomarker protein present in sputum samples of TB infected individuals [9]. Lavania et al. described the use of a HspX specific aptamer for the development of an Aptamer Linked Immobilized Sorbent Assay (ALISA) and an Electrochemical Sensor (ECS) [9]. This study showed that the ALISA and ECS assay with specificities of 94.1% and 91.2%, respectively was significantly better than the conventional Antibody Linked Immobilized Sorbent Assay, which had a specificity of 68.2% [9]. Another aptamer-based biosensor assay which targeted MPT64 employed aptamers coupled to gold electrodes [133]. This study reported a limit of detection of 81 pMol for MPT64 and a reduction in the assay time from days/hours to 30 min.

### 1.7. Potential Biomarkers for Rapid Detection of TB

A good TB diagnostic biomarker should be a detectable molecule that is either exclusively expressed by *M. tb* or differentially expressed by the *M. tb*, or host molecules that are differentially expressed in response to infection by *M. tb* [134]. A number of *M. tb* proteins have been identified in various tissues of TB patients and are potential new biomarkers for the development of more specific diagnosis tests for TB. Some of these potential biomarkers will be discussed in this review.

#### 1.7.1. Blood-Associated TB Biomarkers

For decades, blood has been the preferred biological fluid used especially in immunoassays for the detection of diseases. Blood is a rich source of biomarkers that can provide insights into pathological and physiological processes that are linked to disease states [135]. Though invasive, the ease of obtaining blood samples from patients makes it ideal for the identification of TB-related biomarkers present in the circulation following *M. tb* infections [136,137,138]. Proteomics can provide a comprehensive assessment of protein profiles in different disease states; information on the expression, localisation, interaction networks and activity of a protein [139]. As such, proteomics can be applied in early diagnosis of diseases, prognosis as well as monitoring disease development [140]. Proteome analysis of serum samples has been critical in identifying several biomarkers associated with the pathophysiology of TB [141,142]. Several differentially expressed TB serum proteins have been identified as biomarkers that can be used to discriminate between active and latent TB infections [143]. These include several cell surface adhesins that aid in the pathogenesis of TB [144,145], as they facilitate bacterial aggregation, cell adhesion and infection to the host cells.

Heparin-binding haemagglutinin adhesin (HBHA), a surface-exposed TB protein, is one of the adhesins of *M. tb*, whose expression can be used as an indicator for PTB [146]. More so, HBHA has been reported to induce overproduction of IFN-γ in latent TB patients [147,148]. Compared to latent TB, the response to HBHA in active TB was reported to be present at low concentrations. This was, however, in contrast to ESAT-6, which was reported to be overexpressed in active TB [148]. Additionally, the lack of HBHA in individuals with latent TB but who are also coinfected with HIV, is an indication of increased chances of developing active TB [148,149]. Therefore, both HBHA and ESAT-6 can be used to discriminate between active and latent TB. In addition, other proteins, such as two hypothetical proteins (MT1560.1 and Rv1597), a conserved hypothetical protein (Rv0049), a fatty-acid-CoA synthetase (Rv0270) and a diacylglycerol acyltransferase (Rv3480c) have been reported to discriminate between active and latent TB [143]. The ability of biomarkers to distinguish between active and latent TB infection is thus crucial for an effective TB diagnosis prior to treatment. Some of the serum-based biomarkers identified from serum samples collected from both HIV^+^ and HIV^−^ TB patients are summarised in Table 4. Eight biomarkers were identified and validated as novel TB biomarkers that can potentially discriminate between TB patients with and without HIV [150].

The four culture filtrate protein (CFP) proteins, including single-strand binding protein Ssb, conserved protein, heat shock protein HspX, and EchA1 were previously identified in TB samples using ELISA tests [151,152,153], luciferase-based immunoassay [154,155], and also in *M. tb* culture filtrates [156]. The conserved protein and HspX were isolated from the bacterial cell wall [157,158], while Ssb was identified in the *M. tb* cell membrane [157,159]. Although the location of the EchA1 remains unknown, it was reported to be involved in the oxidation of fatty acids [152,153] and induction of strong antibody responses in both HIV^+^ and HIV^−^ TB patients [152]. In addition, the gene that encode for EchA1 protein was only present in *M. tb* and absent in all 13 BCG strains as well as *M. bovis*, making it a highly specific biomarker [152,153].

The possible transcriptional regulatory protein, a member of the TetR family of transcriptional regulators (TFTRs) involved in antibiotic resistance, was identified as TB drug target using bioinformatics [160]. The TFTR proteins have a conserved helix-turn-helix (HTH) motif in their N-terminal region and a divergent ligand-binding regulatory domain in its C-terminus. The HTH motif binds to palindromic (inverted repeat) sequences of DNA [161,162] and could aid in the regulation of genes in close proximity [163,164].

ACAD/fadE28, Chorismate mutase, PE-PGRS family protein PE_PGRS48, and possible transcriptional regulatory protein do not trigger immune responses as they have been reported to resist and manipulate the complex host immune system [95,165,166]. ACAD/fadE28, is a dehydrogenase protein that forms a heteromeric complex with fadE29 protein catalysing the dehydrogenation of host lipids, such as cholesterol, thereby supporting the survival of *M. tb* during infection [167]. Inactivation of ACAD/fadE28 genes was reported to reduce the survival rate of *M. tb* inside macrophages of patients and mice [168,169].

Recently, several TB-specific biomarkers were identified from the blood samples of *M. tb* infected patients. The proteins included anthranilate synthase component II (TrpG, Rv0013), alanine racemase (Alr, Rv3423c), maltooligosyltrehalose synthase (TreY, Rv1563c), bacterioferritin (BfrA, Rv1876), and conserved hypothetical protein (EspR, Rv3849) [170]. TrpG is an ideal target for anti-TB drugs [171], as it catalyses the biosynthesis of tryptophan, which is essential for the infection and survival of *M. tb* [172,173,174]. However, TrpG is a non-specific biomarker as it is present in all the mycobacterial species [173]. Alr belongs to a class of enzymes known as racemases that catalyse the reversible racemization of L- and D-alanine, an essential component of the peptidoglycan layer found in Gram negative and positive bacteria [174]. TreY protein is also an enzyme involved in starch and sucrose metabolism, specifically trehalose biosynthesis [175]. Trehalose, a disaccharide, is a key component of various glycolipids vital for *M. tb* growth and virulence [176,177,178]. TreY is thus a key component of numerous glycolipids required for the growth and virulent nature of *M. tb* [175] and it is essential for lipid metabolism and mycobacterial cell wall maintenance [179]. On the other hand, BfrB protein, which is similar to mycobactin (MBT) protein, is a sidophore enzyme released by *M. tb* to promote its survival in the host [134,171]. BfrB is a heme binding bacterioferritin, and similar to BfrA, is an iron storage protein unique to *M. tb* [180,181]. BfrB is useful in detecting and storing iron in low iron conditions, while BfrA stores iron in environments with excessive iron levels [180,181]. BfrB also contributes to the virulence of *M. tb* by promoting the bacterium’s intracellular survival and replication [181]. EspR protein is an extracellular transcriptional regulator associated with bacterial cell wall function as well as the regulation of multiple genes such as the espACD operon which is a key component of *M. tb* type VII secretion (TSS7) of the ESX-1 system [182]. The ESX-1 system is responsible for the production and secretion of ESAT-6 and CFP-10 [183,184]; the cell wall proteins involved in various cellular processes [185].

#### 1.7.2. Urine-Associated TB Biomarkers

Urine is an ultra-filtrate of blood and contains molecules that originated from all organs of the body. Eleven potential TB biomarkers were identified in urine samples collected from TB patients and are shown in Table 5. Ten of these proteins were from South African patients with active PTB, with no indication of major comorbidities. Eight proteins were *M. tb*-specific biomarkers, while aconitate hydratase and conserved proteins have been identified in other *Mycobacterium* species [186]. None of these biomarkers are currently used in clinical tests, except LAM.

Despite the unknown function of PE-PGRS protein of 1661 amino acids and PE-PGRS protein of 1307 amino acids, they have been associated with the bacterial cell envelope and are probably involved in host-immune responses and bacterial aggregation [179,186,188,189,190,191]. The *PE-PGRS* genes are characterized by a high G/C-rich repetitive region. These repetitive units are prone to elevated rates of mitotic and meiotic recombination that may alter the antigenic properties of the proteins and contributes to the immune evasion potential of the bacteria during its infectious cycle [192]. Serine/threonine protein kinase transmembrane proteins is involved in different cellular functions which include protection of the bacteria during stress, regulation of the cell cycle and cellular development [193,194]. DNA translocase FtsK; also, a transmembrane protein, belongs to the ATPase family and is involved in the translocation of DNA and proteins through membrane-spanning pores [195]. Single cytoplasmic ATPase domains have also been reported to form homohexameric rings that create pores with a central channel large enough to allow the passage for the double-stranded DNA substrate during the infectious cycle [196]. The respiratory nitrate reductase (NarG, Rv1161), an enzyme that is involved in nitrogen metabolism and catalyses the consumption of nitrate in *M. tb*, was identified during latent TB infections [170]. This was contradictory to the study by Young and colleagues, who reported that NarG is associated with active TB infections [186]. Regardless of these contradicting evaluations, NarG is known to contribute towards the virulent nature of *M. tb* and promote the anaerobic growth of the bacterium on glycerol [197,198]. This protein is also conserved in other *Mycobacterium* species and is dependent on the molybdopterin cofactor to carry out its function [199]. It is also upregulated in the presence of nitrate during the dormant stages of *M. tb* [200].

The probable dehydrogenase protein is encoded by the *RV2280* gene which was shown to be linked with *PE-PGRS* genes. The *RV2280* gene was reported to be upregulated by the *IS6110* and *IS110* genes. The *IS110* gene may directly influence the recognition of *M. tb* by the immune system through insertional inactivation and upregulation of *PE-PGRS* genes [201,202]. The *RV2280* gene was also predicted to encode for a 459 amino acid protein [192] which was closely related to a family of FAD/FMN-containing dehydrogenases and FAD-linked glycolate oxidases [201]. Other studies also reported that the Rv2280 protein contains a potential binding domain for FAD cofactor and, like other members of this family, may be involved in energy metabolism [203,204]. However, the effect of upregulating the *Rv2280* gene still remains unknown. The conserved protein is a membrane-associated protein that was identified from Triton X-114 extracts of *M. tb* H37Rv strain [123] and in membrane protein fractions and whole cell lysates of *M. tb* [156] using mass spectrometry. The protein plays a role in a pathway that allows the adaptation of *M. tb* to stressful conditions caused by hypoxia and antibiotics [205]. The polyketide synthase protein on the other hand is a cytoplasmic protein [206], with unknown bacterial function [207].

LAM is a protein that is used to screen patients who have clinically manifested HIV/AIDS symptoms and more severe clinical disease or who are at greater risk of mortality [208]. It was also detected in urine samples collected from HIV^+^ TB patients [187]. LAM is a glycolipid present within the cell wall of *M. tb*. and can be used as a potential biomarker in the identification of active HIV-associated TB infections. The LAM screening test is considered relatively inexpensive (USD 2.66/test) and could be performed readily to identify LAM proteins in cases of HIV-associated PTB [209,210]. In addition, the test could be used to monitor a patient’s response to anti-TB therapy in resource-limited HIV-endemic settings [211]. Regardless of these positive aspects, the LAM test was associated with some limitations such as limited sensitivity in diagnosing HIV^+^/TB co-infections [187] and the fact that LAM protein concentrations in urine samples of HIV positive TB patients is very low [212]. Further studies are required to determine whether a persistent positive LAM urine test is due to drug-resistant TB, poor medication, or absence of a therapeutic response [211].

#### 1.7.3. Multi-Target TB Biomarkers

Two of the *M. tb* secreted small molecules, mycobactin T (MBT, Rv2895c) and 1-tuberculosinyl adenosine (TbAd) have been identified in PTB patients using a single liquid chromatography–tandem mass spectrometry. These small molecules showed 100% specificity to *M. tb* [213]. MBT and TbAd were released by *M. tb* into the blood, sputum, cerebrospinal fluid (CSF), lung, and lymph nodes of TB patients, and in serum and lungs of BALB/c mice infected with the virulent *M. tb* H37Rv strain [213]. MBT proved to be a better biomarker than the TbAd and was detected in ≥40% patients. The detection of these two molecules in human CSF and lymph node tissues could substantiate their use as *M. tb*-specific biomarkers.

TbAd is a highly abundant lipid-linked nucleoside that comprises a major class of lipids in *M. tb.* It was identified only in patients infected by *M. tb* unlike other extracellular proteins such as MPT64 (Rv1980c) and MPT51 (Rv3803c) which were identified in both *M. tb* and *M. bovis* [214,215]. *M. tb* and *M. bovis* are evolutionarily related and share more than 99% sequence identity. However, only *M. tb* expresses TbAd and causes widespread infections [215]. In addition to its specific expression, the high abundance and shedding of TbAd from intact *M. tb* in ways that permit in vivo detection makes TbAd an ideal TB diagnostic marker [215].

MBT is a cell-wall associated sidophore (iron chelator) produced by *M. tb* to promote its survival in the host [216,217]. Iron is an essential nutrient for most living organisms including *M. tb*. Due to the poor solubility of ferric iron (Fe^+3^) in aerobic neutral pH environments, free iron is absent in mammalian hosts but is sequestered in protein complexes such as transferrin, lactoferrin and ferritin [218]. In addition, the host macrophages create an iron-limiting environment for *M. tb* [219,220]. The ability to acquire free iron within hosts during infection cycles is thus a serious challenge for most pathogens. To overcome this, *M. tb* synthesizes and secretes high affinity iron chelators (siderophores), such as MBT, to solubilize iron and efficiently compete with the hosts’ iron-binding proteins. The MBT protein acquires and transports iron from the periplasmic space to the plasma membrane-bound protein complex (iron-regulated transporters) [219,221]. The potential use of TbAd and MBT in the diagnosis of active TB infections could offer a first-line approach towards TB control consequently leading to early therapeutic interventions.

## 2. Conclusions

An increase in TB infections and related deaths have exposed the urgent need for the development of rapid TB-specific diagnostic screening assays that will be more efficient, affordable, and accessible. Recent advances in the identification of new TB biomarker proteins that can differentiate between latent and active TB, as well as biomarkers that can distinguish between TB infections and other respiratory infections can significantly improve the diagnosis of TB. Potential TB biomarkers have been identified in urine, CSF, and blood samples of TB patients; some were validated clinically for TB diagnosis. Current tests (TST and IGRAs), together with other clinically used diagnostic approaches, are time-consuming (culture and sputum smear tests), and expensive (GeneXpert). These tests cannot be used for routine rapid TB screening in resource limited environments. The rapid immunoassays use antibodies that have low sensitivity and stability. Great improvement has been observed when aptamers are used instead of antibodies in rapid diagnostic tests such as LFAs. The application of aptamers as recognition elements for the detection of newly identified biomarkers and the revelation of more specific TB biomarkers can in the future deliver POC TB diagnostic devices that are, rapid, cost-effective, and highly sensitive. Developing such diagnostic tools would be beneficial to resource-limited countries that cannot afford expensive diagnostic laboratories. In addition, these tests would allow for short diagnostic turnaround time and potentially expedite the treatment process. Therefore, this review highlighted that, the application of aptamers in the rapid screening of TB infections hold promise to the global reduction of the infections in the near future and this will be in line with WHO’s ASSURED requirement.

## Figures and Tables

**Table 1:** Comparison between QFT-GIT and T-SPOT tests [73].

Feature	QFT-GIT	T-SPOT
Test sample	Whole blood	Peripheral blood mononuclear cells (PBMCs)
*M. tb* antigens tested	Single peptides such as the early secretory antigenic target 6 (ESAT-6) Culture filtrate protein 10 (CFP-10)	Can detect one or both peptides (ESAT-6 and CFP-10)
Measurement	IFN-ƴ concentration.	Number of IFN-ƴ producing cells
Possible Results	Positive, negative, indeterminate	Positive, negative indeterminate, borderline
Processing time	Takes about 24 h	Processes within 8 h

**Table 2:** Properties of current TB diagnostic tests.

Diagnostic Test	Test Sample	Targets	Specificity	Sensitivity	Cost (USD Per Test)	Turn-Around Time	References
Sputum smear microscopy	Sputum	Bacilli	99.1%	50–60%	13.31–99,350	1 h	[59,60,61,97]
Sputum culture	Sputum	*M. tb*	98%	>80%	15–143,432	4 weeks	[59,60,61,97]
Chest X-ray and Ultrasonography	Lungs	Lesions in the lungs	>68.6%	>76.4%	7.8—672,298	<30 min	[65,66,68,69]
TST	Skin	Tuberculin	88%	94%	3–13	48–72 h	[70,98]
IGRA	Blood	IFN-ƴ	76.37%	76.66%	46.61—55.08	8–24 h	[72,73,74,99]
GeneXpert	Sputum	*M. tb* DNA	100%	98.6%	252,876	2 h	[75,77,78,97]
Line probe assay	Sputum	*M. tb*	99.3%	96.9%	107,212	7 h	[97,100]
Serological tests	Blood	*M. tb* antibodies and nucleic acids	84–100%	43–71%	10–30	<30 min	[93,94,95]

Note: The costs are based on rates from 2015–2018.

**Table 3:** Comparison of the properties of aptamers to antibodies (adopted from [96,111]).

Properties	Antibody	Aptamer
Production	Time-consuming (weeks-months)	Chemical synthesis (1–2 days, such as capillary electrophoresis-based SELEX)
Selection	Limited to animal immunisation	In vitro and in vivo selection under a variety of conditions
Oriented immobilization	Difficult through protein A/G	Easy through various chemical modifications
Target	Proteins or haptens. Difficult for non-immunogenic or toxic targets	Any targets from ions to whole cells, including non-immunogenic or toxic target
Modification	Difficult and expensive to modify	Cheap and easy to modify with other active groups in a large scale
Shelf life	Short shelf life and require a continuous cold storage	Long shelf life and does not require special storage conditions
Stability	Sensitive to pH and temperature	Tolerant of pH and temperature
Cost	1 mg of a modified antibody costs USD ~1000	1 mg of a modified aptamer costs USD ~100

**Table 4:** TB biomarkers identified in sera from HIV^−^ and HIV^+^ TB patients.

Biomarkers	Locus/Accession No.	HIV Status	References
Single-strand binding (Ssb) protein or helix-destabilizing protein	^a,b^Rv0054	+	[150]
Chorismate mutase	^a^Rv0948c	+	[150]
Heat shock protein HspX	^a^Rv2031c	-	[150]
Conserved protein	^a,b^Rv0831c	0	[150]
Possible transcriptional regulatory protein	^b^Rv3405c	+	[150]
PE-PGRS family protein PE_PGRS48	^a^Rv2853	-	[150]
Acyl-coenzyme A dehydrogenase (ACAD)	^b^Rv3544c	0	[150]
Enoyl-CoA hydratase, EchA1	^b^Rv0222	0	[150]
HBHA	^c^MT18B_0591	-	[148,149]
ESAT-6	^c^Rv3875	+	[148,149]

^a^ protein identified in SA, ^b^ in USA, and ^c^ in Italy patients, ^0^ not known.

**Table 5:** *M. tb* proteins identified in human urine samples from active TB patients.

Protein Name	Accession #	*M. tb* Specific	Reference
Aconitate hydratase	Rv1475c	X	[186]
Conserved protein	Rv1977^+^	X	[186]
Serine/threonine protein kinase	Rv0014c	✓	[186]
DNA translocase FtsK	Rv2748c	✓	[186]
Nitrate reductase α-subunit	Rv1161	✓	[186]
Uncharacterised FAD-linked oxidoreductase	Rv2280	✓	[186]
Conserved hypothetical protein	Rv2694c	✓	[186]
Polyketide synthase	Rv1664	✓	[186]
PE-PGRS protein of 1661 amino acids	Rv2490c	✓	[186]
PE-PGRS protein of 1307 amino acids	Rv0578c	✓	[186]
Lipoarabinomannan (LAM)	Rv2188c Rv2181	X	[187]

Note: X—Non-specific, ✓—Specific.

## Data Availability

Not applicable.
